# PET in neurotherapeutic discovery and development

**DOI:** 10.1016/j.neurot.2024.e00498

**Published:** 2024-12-10

**Authors:** Melissa Chassé, Neil Vasdev

**Affiliations:** a Azrieli Centre for Neuro-Radiochemistry, Brain Health Imaging Centre, Centre for Addiction and Mental Health, Campbell Family Mental Health Research Institute, Toronto, Canada; b Institute of Medical Science, Temerty Faculty of Medicine, University of Toronto, Canada

**Keywords:** Positron emission tomography, Neuroimaging, Radiochemistry, Small molecule drug development, Carbon-11, Fluorine-18

## Abstract

Positron emission tomography (PET) is a highly sensitive, quantitative imaging technique that can track sub-nanomolar quantities of positron-emitting radionuclides throughout the body. By incorporating such radionuclides into molecules of interest, we can directly assess their pharmacokinetic and pharmacodynamic (PK/PD) characteristics *in vivo* without changing their physicochemical characteristics or eliciting a pharmacological response. As such, PET imaging has long been used as a tool to aid drug discovery programs from preclinical biomarker validation all the way through to clinical trials. In this perspective we discuss the use of PET radioligands in central nervous system (CNS) drug discovery and development, with a focus on recent applications in psychiatry (e.g. 5-HT_2_A, 11β-HSD1), neuro-oncology (e.g. KRAS^G12C^, ATM, ALK2), and neurodegeneration (e.g. amyloid beta plaques, MAPK p38), while exploring the intricacies associated with developing novel radiotracers for CNS targets. Examples highlight the preclinical and clinical uses of PET for studying biomarker function, drug candidate PK/PD, target occupancy/engagement, dosing regimen determination, clinical trial patient selection, and quantifying biomarker changes in response to treatments.

## Introduction

Central nervous system (CNS) disorders constitute a major global health challenge. It is estimated that over 40 ​% of the population experienced CNS health loss in 2021, including neurodevelopmental disorders, neuro-oncology, age-related neurodegeneration, and psychiatric conditions [1]. Mental health conditions alone had an estimated 970 million cases in 2019, a near 50 ​% increase compared to 1990 [2]. Despite this growing epidemiological crisis, novel CNS drug development has slowed over the past few decades [[3], [4], [5]] because many large pharmaceutical companies divested from their CNS drug discovery pipelines in the 2010s [3] as a result of the fiscal challenges and high attrition rates associated with neurotherapeutic development.

Preclinical development for CNS drugs is notoriously difficult because the novel molecular entities must not only demonstrate efficacy and minimal off-target effects, but they must also contend with surpassing the blood-brain barrier (BBB) in sufficient levels to be therapeutically relevant [[6], [7], [8], [9], [10], [11]]. Even once a company has a lead candidate after screening up to millions of compounds in the preclinical hit-to-lead process [6], 80–90 ​% of Phase I candidates do not make it through regulatory approval [[12], [13], [14]]. CNS drug development suffers from one of the highest rates of late-stage failure, with nearly half of all CNS drugs in Phase III being discontinued due to lack of efficacy [[12], [13], [14]]. Estimates place the timeline and budget for advancing a drug candidate to Phase III clinical trials in the range of 10–20 years and millions-to-billions of US dollars [6,15]. Given the cost and high attrition rates, tools that are able to aid and de-risk the neurotherapeutic R&D process are in high demand.

Positron emission tomography (PET) is a medical imaging technique that can be used to de-risk CNS drug discovery and development [16,17]. Molecules containing positron-emitting radionuclides, known as radiotracers, are designed to target a biochemical process or specific protein (e.g. receptor, enzyme, protein aggregate, etc.). As the radiotracer travels through the body, the integrated radionuclides undergo radioactive decay, releasing a positron (β^+^) which annihilates on contact with an electron to create two 511 ​keV photons traveling approximately 180° apart. These coincident photons are then detected by the scanner for reconstruction into a three-dimensional image. By creating PET-isotopologs of lead drug candidates, their pharmacokinetics and pharmacodynamics can be evaluated in vivo while maintaining their physicochemical characteristics; this includes key CNS drug factors like brain uptake/washout rates, drug-target engagement, target occupancy, drug metabolism, and excretion routes. Moreover, PET radiotracers are administered at sub-pharmacological doses, and are ideal for translation to non-human primates (NHPs) or human use without eliciting a pharmacological response or risking adverse effects.

**Fig. 1:**
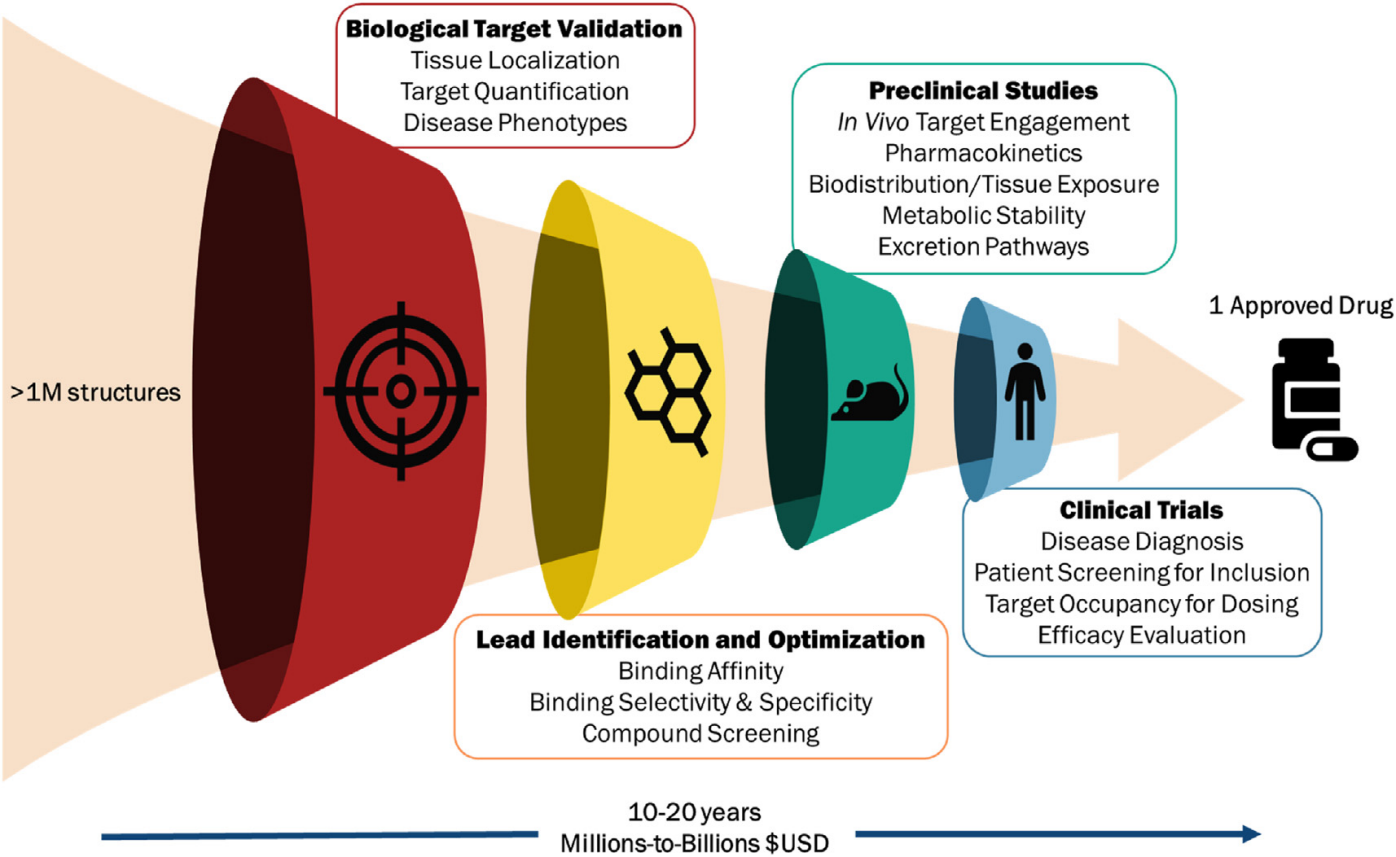
Graphic depicting the drug discovery process, in which millions of chemical structures are eventually narrowed down to one approved drug – a costly and time-consuming process. The numerous ways in which molecular imaging can be applied to aid attrition rates and inform drug development at various stages are shown.

In this perspective article, we offer an overview of how PET radiotracers can be applied to neurotherapeutic programs of all stages, with an emphasis on recent literature examples in the fields of neuropsychiatry, neurodegeneration, and neuro-oncology. This current perspective article is intended to provide a short, updated view on the multifaceted uses of CNS PET in selected examples of neurotherapeutic development (Fig. 1).

### Serotonin 5-HT_2_A receptor (5-HT_2_AR)

**Fig. 2:**
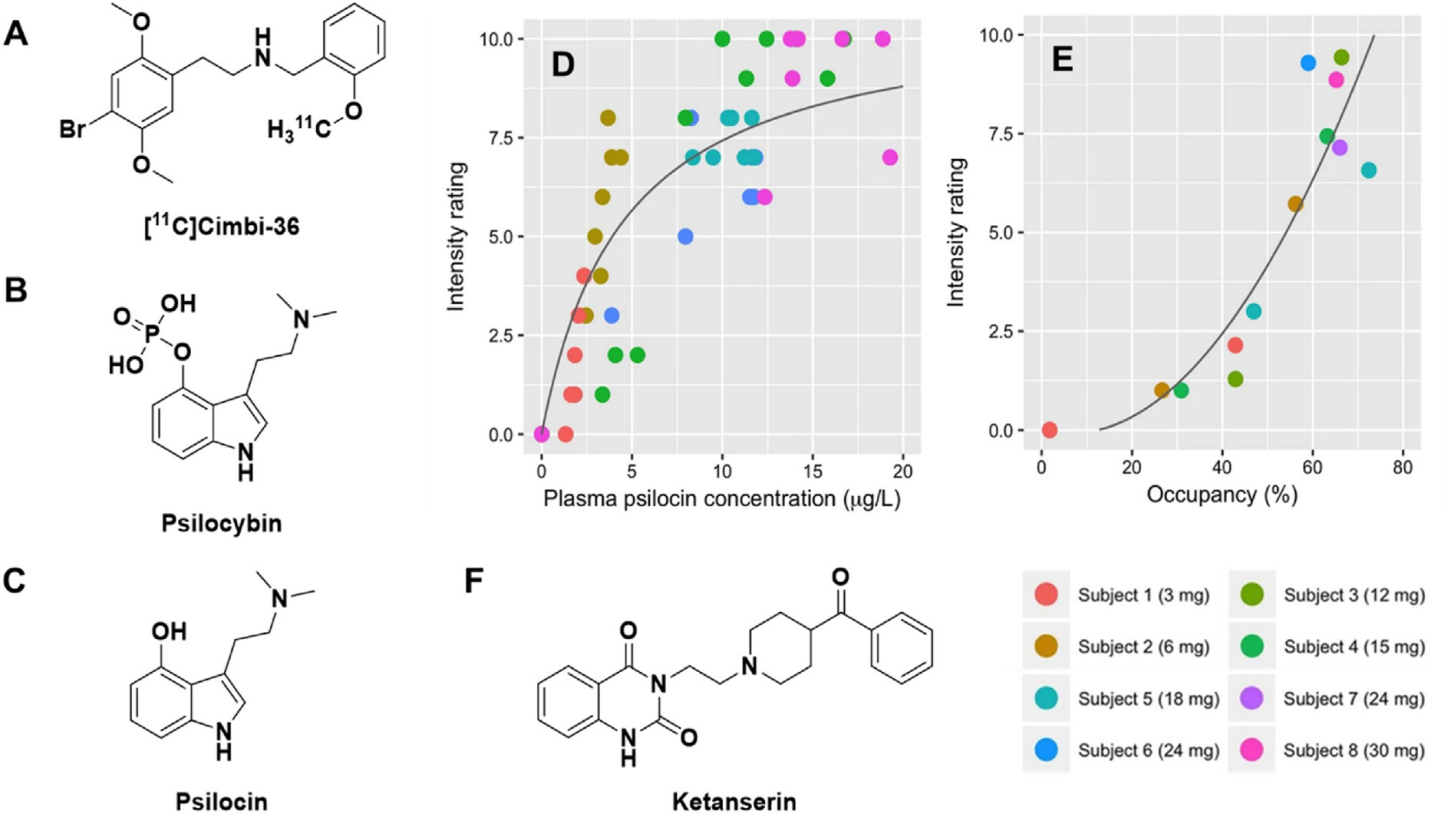
(A) Structure of [^11^C]Cimbi-36. (B) Relationship between subjective intensity rating after psylocibin dosing and neocortical 5-HT_2_AR occupancy. (C) Relationship between subjective intensity rating and plasma psilocin concentration. Adapted from Madsen MK et al. Psychedelic effects of psilocybin correlate with serotonin 2A receptor occupancy and plasma psilocin levels. Neuropsychopharmacol 2019; 44:1328–1334. Springer Nature.

The popularity of psychedelic therapy has skyrocketed in recent years, with ongoing investigations into its potential for treating conditions including anxiety disorders, post-traumatic stress disorder, substance use disorders and treatment resistant depression [[18], [19], [20]]. Psychedelic effects are thought to arise from stimulation of serotonin 5-HT_2_A receptors (5-HT_2_AR) [21], making this target of particular interest for studying the origins of phenomena such as sensory hallucination, and assessing whether these affects can (or should) be detangled from the potential benefits to mood and cognitive processes. Neuroimaging has been used extensively in this psychedelic renaissance for quantifying neurochemical changes associated with psychedelic therapy [22,23]. In particular, we highlight the role that [^11^C]Cimbi-36 (Fig. 2A) has played in studying target occupancy and the role of 5-HT_2_AR in psychedelic research.

[^11^C]Cimbi-36 is a 5-HT_2_AR agonist radioligand with quantifiable off-target affinity for serotonin 5-HT_2_C receptors [[24], [25], [26]]. [^11^C]Cimbi-36 has been used to investigate 5-HT_2_AR target occupancy for several molecules with known serotonergic activity. Notably, this radiotracer was used to assess the relationship between the subjective psychedelic effects of psilocybin, plasma levels of its active metabolite psilocin, and 5-HT_2_AR occupancy in healthy human volunteers [27] (Fig. 2B–E). This study found a dose-dependent association of psylocibin ingested (3–30 ​mg) and plasma psilocin with cerebral 5-HT_2_AR occupancy, reporting an EC_50_ for psilocin of 1.95 ​μg/L (or 10 ​nM). Moreover, neocortical target occupancy was shown to positively correlate with subjective psychedelic effect intensity ratings. Interestingly, these results support a range of 0.5–2 ​mg psylocibin for micro-dosing in psychedelic therapy, as that range should correspond with low target occupancies and, thus, low subjective psychedelic effect scores. These target occupancy PET studies have contributed to our current understanding of psylocibin's mechanism of action, while also providing important information for clinical trials in psychedelic therapy.

A key concern about using psychedelic treatment for psychiatric conditions is the potential for unpleasant psychedelic effects which may cause the patient significant distress and/or exacerbate the underlying psychiatric condition. To this end, a rescue drug to stop or shorten psychedelic effects could be a useful treatment tool. Ketanserin is a 5-HT_2_AR specific antagonist known to prevent and/or terminate the subjective effects induced by certain psychedelics when used as a pre-treatment or rescue drug (Fig. 2F) [[28], [29], [30], [31]]. The selectivity of ketanserin for 5-HT_2_ARs may provide an advantage over the current antipsychotic methods of shortening distressing psychedelic experiences that are attributed to off-target affinity for dopamine D_2_ receptors [30,31]. The 5-HT_2_AR target occupancy of ketanserin was measured using [^11^C]Cimbi-36, which yielded information pertinent to its potential use as a psychedelic rescue drug [32]. The study found that orally administered ketanserin (10–40 ​mg) had a dose-dependent cerebral 5-HT_2_AR occupancy with an EC_50_ of 2.52 ​μg/L (or 6.4 ​nM). These findings suggest that selective 5-HT_2_AR antagonists or inverse agonists may be preferable alternatives to the current antipsychotics being used as rescue medications for bad psychedelic experiences, while also providing neurochemical rationale that strongly supports previous rescue drug dosing regimen and drug rescue kinetics studies.

While the ongoing research in psychedelic therapy is certainly exciting, legal restrictions on controlled substances continue to hamper these studies globally. As such, readers should note that regulatory approvals and security compliance may be required to conduct psychedelic therapies and/or radiopharmaceutical production in certain jurisdictions.

### 11β-Hydroxysteroid dehydrogenase type 1 (11β-HSD1)

11β-Hydroxysteroid dehydrogenase type 1 (11β-HSD1) is an intracellular enzyme responsible for reducing cortisone to glucocorticoid cortisol, and is widely expressed in adult prefrontal cortex, hippocampus, and cerebellar tissues [33,34]. Brain cortisol levels regulate the hypothalamic-pituitary-adrenal (HPA) axis, a major part of the neuroendocrine system that controls stress responses. Irregular cortisone metabolism has been associated with numerous psychiatric conditions, including depression, post-traumatic stress disorder (PTSD), and substance use disorders, as well as age-related cognitive decline [[33], [34], [35], [36], [37], [38]]. Several recent clinical trials have investigated 11β-HSD1 inhibitors in CNS conditions (NCT02727699; NCT01146886; NCT01146886; NCT05657691; NCT02017444).

**Fig. 3:**
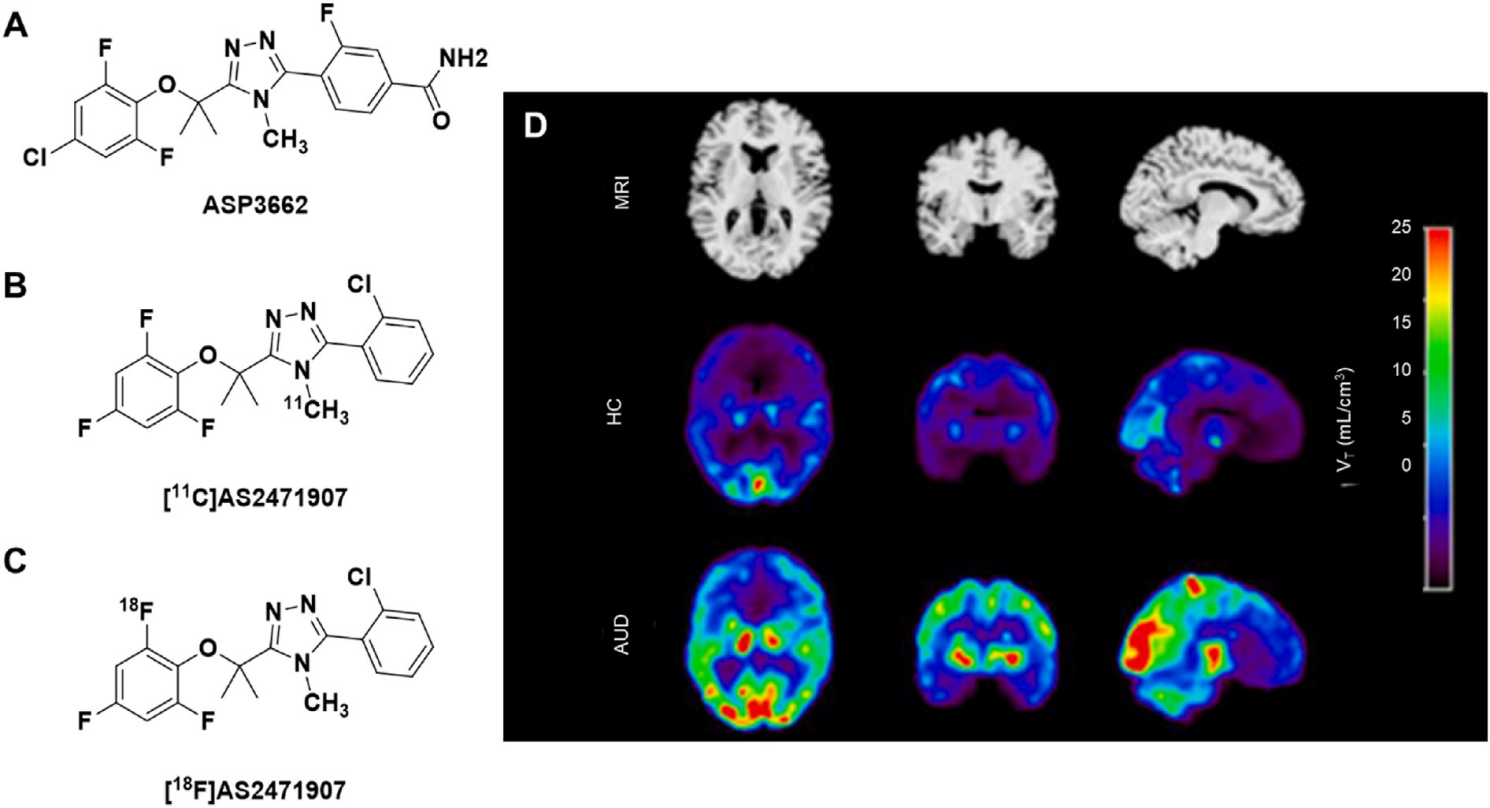
Structures of 11β-HSD1 radiotracers (A) [^11^C]AS2471907 and (B) [^18^F]AS2471907. (C) Participant's MRI and co-registered parametric [^18^F]AS2471907 *V*_T_ images in age-matched and sex-matched individuals with alcohol-use disorder (AUD) versus healthy control (HC). Adapted from Verplaetse TL et al. Imaging a putative marker of brain cortisol regulation in alcohol use disorder. Neurobiol Stress 2024; 29:100609. Copyright (2024), with permission from Elsevier.

One clinical trial that made use of PET imaging was sponsored by Astellas Pharma, investigating the pharmacokinetics, pharmacodynamics, and safety profile of their 11β-HSD1 inhibitor, ASP3662, at single and multidose regimens in healthy volunteers (NCT02194491) (Fig. 3A). Originally, preclinical pharmacology data was used to estimate starting doses for humans; however, this led to a large range of 0.15 ​mg up to 10 ​mg [39]. Instead of adhering to this estimate, researchers turned to PET imaging to aid their clinical trial study design. Previous enzyme occupancy studies in NHPs with the 11β-HSD1 PET imaging agent [^11^C]AS2471907 (Fig. 3B) predicted that 1 ​mg would inhibit between 30 ​% and 50 ​% of 11β-HSD1 activity in the brain [40,41], leading researchers to use this as their starting dose for the Phase I clinical trial. Human PET data was also collected to determine drug-target engagement within the brain and evaluate maximal enzyme inhibition levels and kinetics, although this imaging data was never published. These studies exemplify how PET can help in early phases of clinical trial design and be used to confirm drug-target interactions in the human brain.

Interestingly, the fluorine-18 labeled isotopolog, [^18^F]AS2471907 (Fig. 3C), is being applied to studying the HPA axis in various aspects of psychiatric research, including recent articles that have shown significantly heightened 11β-HSD1 expression in prefrontal limbic brain regions in PTSD and substance use disorders compared to healthy controls (Fig. 3D) [36,37,42,43]. In this manner, PET imaging is also being used as a putative marker of brain cortisol regulation to elucidate the involvement of 11β-HSD1 in psychiatric conditions that are not currently within the scope of ongoing clinical trials, but which have potential to benefit from 11β-HSD1-related neurotherapeutic intervention in the future.

### Mutant GTPase KRAS^G12C^

Brain cancers have among the lowest survival rates of any cancer [44,45]. The blood-brain barrier is a highly selective endothelial barrier which restricts the passage of chemotherapeutics from peripheral blood flow to CNS fluids, thereby precluding the use of many peripherally active chemotherapeutics. Even if chemotherapies are able to pass the blood-brain barrier, many primary CNS tumors exhibit inherent multidrug resistance. Common cell-cycle checkpoint deficits and activated DNA damage response pathways also contribute to treatment resistance against radiotherapy and chemotherapy [45]. These overlapping resistance mechanisms contribute to poor prognoses for primary and secondary CNS tumors, and a desperate need for improved treatment methods.

**Fig. 4:**
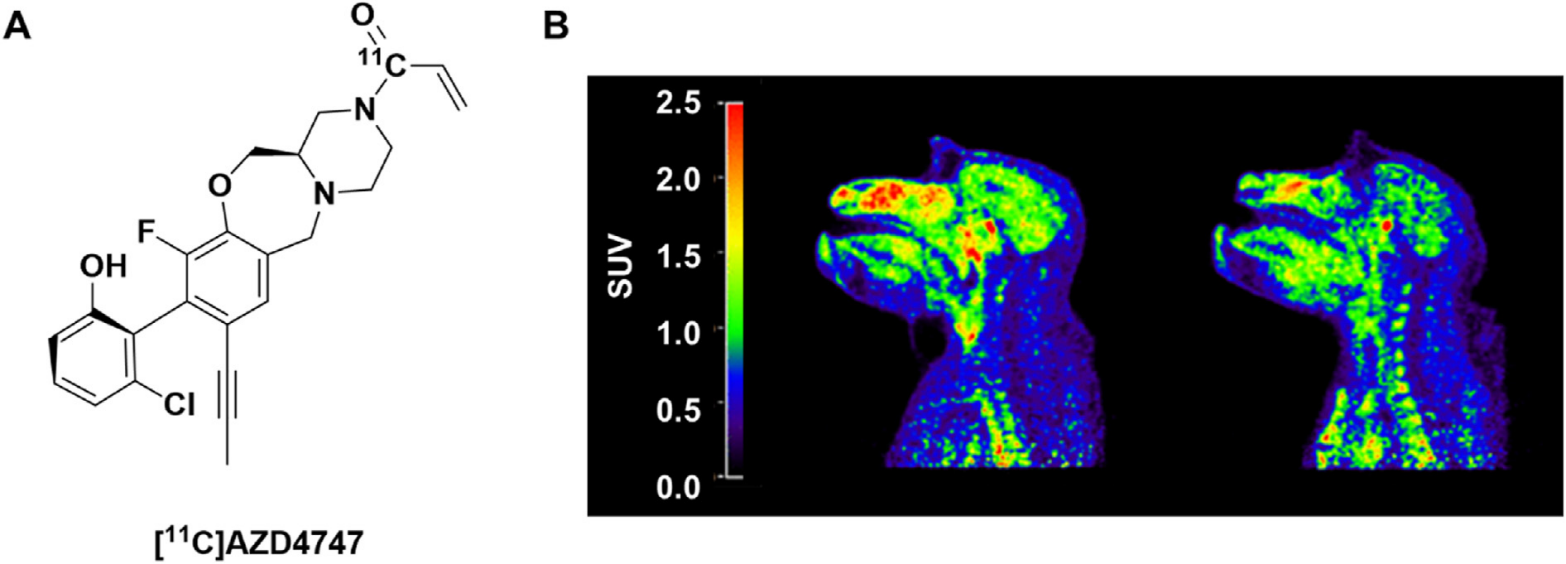
(A) Structure of radiolabeled [^11^C]AZD4747. (B) [^11^C]AZD4747 PET images (average SUV 5–123 ​min) in two cynomolgus monkeys. Adapted with permission from Kettle, J.G. et al. Discovery of AZD4747, a Potent and Selective Inhibitor of Mutant GTPase KRASG12C with Demonstrable CNS Penetration. J Med Chem 2023; 66(13):9147–9160. Copyright 2023 American Chemical Society.

The *KRAS* gene is one of three members in the rat sarcoma viral oncogene family (Ras) and encodes for the KRas (Kirsten RAS) GTPase enzyme. Oncogenic mutations in Ras proteins are found in up to 30 ​% of human cancers, with KRas being the most commonly mutated isoform [46]. Recently, researchers at AstraZeneca discovered a potent inhibitor selective for the glycine to cysteine mutation at codon 12 of the K-Ras GTPase (KRAS^G12C^) [47]. It was hypothesized that this inhibitor could be used as a chemotherapy for secondary brain metastases if it demonstrated sufficient CNS exposure. Preliminary rodent studies demonstrated a mean brain *K*_*pu,u*_ ​= ​0.1, which could be elevated to a K_pu,u_ of 1.0 when co-administered with a P-glycoprotein inhibitor, thereby raising concerns over potential active drug efflux in vivo. By radiolabeling the lead drug compound with carbon-11 ([^11^C]AZD4747, Fig. 4A), the researchers were able to perform a “micro-dosing study” in NHPs to safely assess its CNS exposure in higher species without the risk of toxic effects. The results demonstrated that the compound was able to permeate through the blood-brain barrier (Fig. 4B), sporting an estimated mean brain *K*_*pu,u*_ ​= ​1.6, and suggesting that this compound might cross the intact human BBB in high enough quantities to be pharmacologically active. This case highlights how PET imaging can be used to safely assess pharmacodynamic properties of candidate neurotherapeutics in higher species.

### Ataxia-telangiectasia mutated kinase (ATM)

**Fig. 5:**
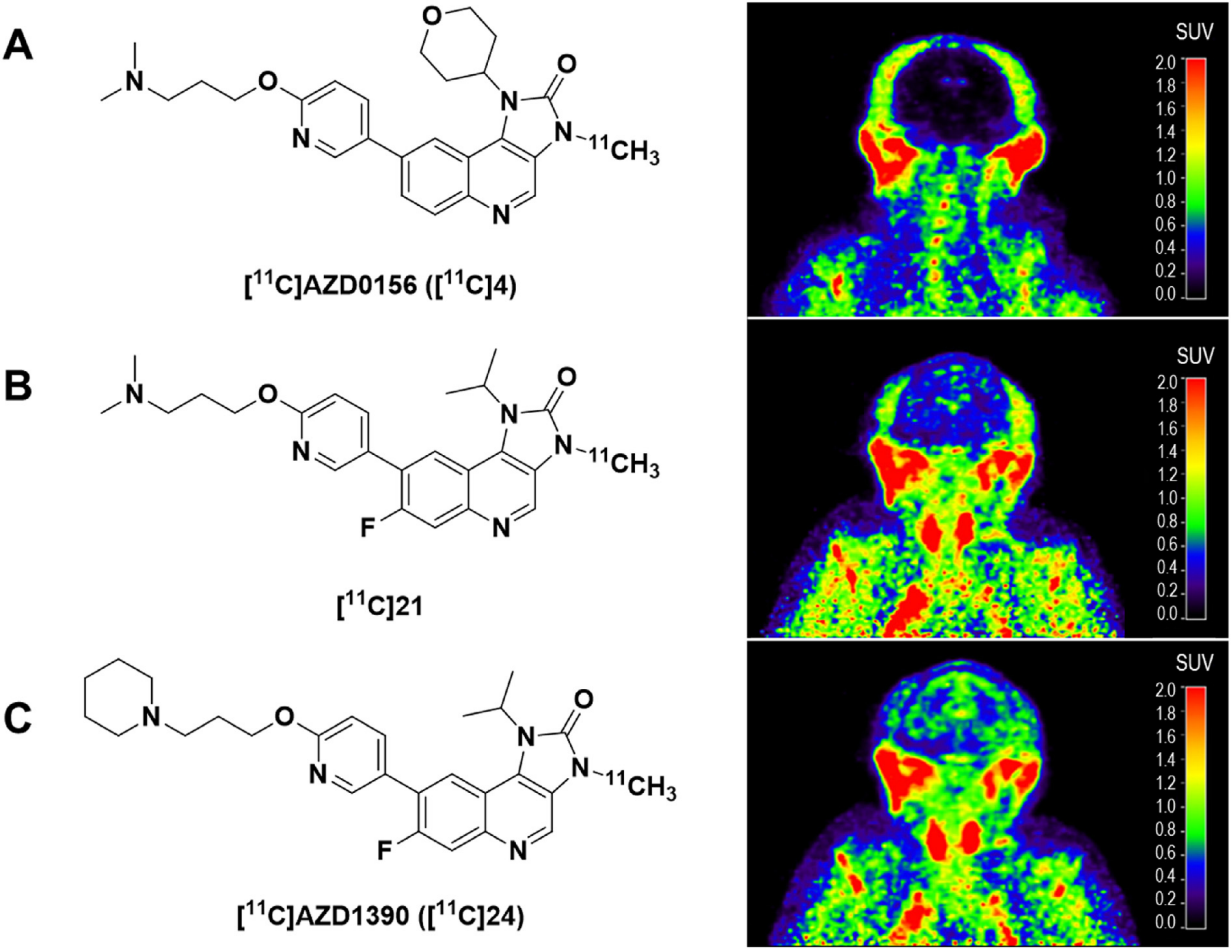
Structures of (A) [^11^C]AZD0156 ([^11^C]4), (B) [^11^C]21, and (C) [^11^C]AZD1390 ([^11^C]24) with their respective summed 5–123 ​min PET images in NHPs. Adapted with permission from Pike KG et al. Identification of Novel, Selective Ataxia-Telangiectasia Mutated Kinase Inhibitors with the Ability to Penetrate the Blood–Brain Barrier: The Discovery of AZD1390. J Med Chem 2024; 66(13):9147–9160. Copyright 2024 American Chemical Society.

A similar study by the same group recently reported on the use of PET for developing brain penetrant ataxia-telangiectasia mutated (ATM) kinase inhibitors [48]. *In vitro* assays have shown that ATM inhibition leads to chemo- and radio-sensitizing effects in human glioma cells, making this target of interest for treating primary brain tumors [49]. In this study, the AstraZeneca ATM inhibitor clinical candidate (AZD0156, Fig. 5A) was tweaked with the intention of improving brain uptake, especially to decrease its known drug efflux activity [48]. After lead optimization, two novel ATM inhibitors were radiolabeled with carbon-11 to assess their CNS exposure compared to the clinical candidate (Fig. 5B,C). Both structures demonstrated promising *K*_*p,uu*_ ​> ​0.3, unlike AZD0156, which showed low brain exposure. Ultimately, AZD1390 was selected for progression based on cell potency, kinase selectivity, and preclinical pharmacokinetic profile. In this case, [^11^C]AZD1390 was pursued even further and assessed in healthy human volunteers, which demonstrated that this novel ATM inhibitor structure is able to cross the intact human blood-brain barrier [50]. These PET studies contributed to the progression of the new clinical candidate AZD1390 into Phase 0/1b (NCT05182905) and Phase 1 (NCT03423628) clinical studies to explore the radiosensitizing effects of ATM inhibition in intracranial malignancies [51].

### Activin-receptor like kinases (ALK)

Another family of proteins that are of interest in neuro-oncology are the activin-receptor like kinases (ALKs) [52]. Seven types of ALKs have been identified in humans (ALK1-7), each with their own biological functions related to development (e.g. vasculogenesis, osteogenesis, chondrogenesis, etc.) and reproductive function. Aberrant ALK performance has been linked to several types of cancers, both as tumor promoters and suppressors. Within the realm of primary CNS cancers, deviations in certain types of ALK performance have been associated with gliomas, glioblastomas, and pituitary cancers. As such, there is much interest in developing chemotherapies for treating primary tumors or metastases within the CNS.

**Fig. 6:**
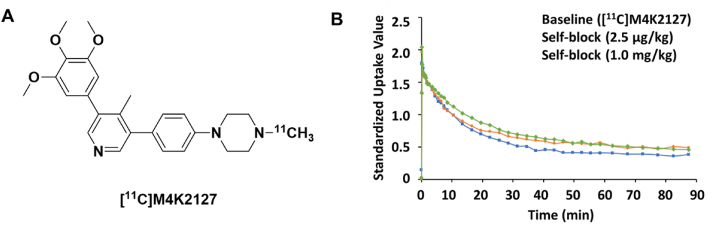
(A) Structure of [^11^C]M4K2127. (B) [^11^C]M4K2127 PET images (average SUV 0–90 ​min) in a healthy rodent. Adapted with permission from Chassé M et al. Leveraging Open Science Drug Development for PET: Preliminary Neuroimaging of ^11^C-Labeled ALK2 Inhibitors. ACS Med Chem Lett 2021; 12:846–850. Copyright 2021 American Chemical Society.

Mutations in the *ACVR1* gene encoding for ALK2 occur in 25–33 ​% of patients with the rare pediatric cancer diffuse intrinsic pontine glioma (DIPG), with many being gain-of-function over-activating mutations [[52], [53], [54]]. Preliminary preclinical studies suggest that ALK2 may be a promising therapeutic target [55], which has led to extensive medicinal chemistry efforts dedicated to developing novel ALK2 inhibitors [[56], [57], [58], [59], [60], [61]]. With no adequate method of determining pons-specific exposure, our laboratories turned to carbon-11 radiolabeling of a few lead 3,5-diphenylpyridine structures for preliminary studies in rodents [62]. The most promising compound appeared to be [^11^C]M4K2127, with high initial brain radioactivity uptake, including homogenous uptake throughout the pons (SUV ∼2) (Fig. 6A). However, subsequent preliminary homologous blocking studies *in vivo* revealed that the observed radioactivity uptake was primarily non-specific binding, with no significant change in brain uptake or washout kinetics at blocking doses of between 0.001 ​mg/kg to 1 ​mg/kg (Fig. 6B) [63]. Radiometabolite analyses showed that only ∼25 ​% of the radioactivity in rodent brain homogenate was intact [^11^C]M4K2127 at 15 ​min post-radiotracer injection; the observed metabolic instability was further supported by poor microsomal stability [57]. This case study demonstrates the utility of PET imaging for characterizing important pharmacokinetic and pharmacodynamic properties of potential drug candidates, including metabolic stability. For PET chemists, it also highlights the importance of fully characterizing a radiotracer's in vivo characteristics, including assessment of potential confounding brain-penetrant radiometabolites [64].

### Mitogen activated protein kinase p38α/β (p38α/β)

The p38α kinase has shown clinical and preclinical promise as a druggable target in several inflammatory conditions, including neurodegenerative disease [65,66]. Adverse effects reported in previous clinical trials using talmapimod for peripheral inflammatory conditions suggested that the inhibitor may be CNS permeable, with reports including dizziness, somnolence, nausea, neuropathy, and headache [[67], [68], [69]]. If this existing clinical trial candidate had significant CNS exposure, it could potentially be repurposed for treating neurodegenerative inflammation at lower doses.

**Fig. 7:**
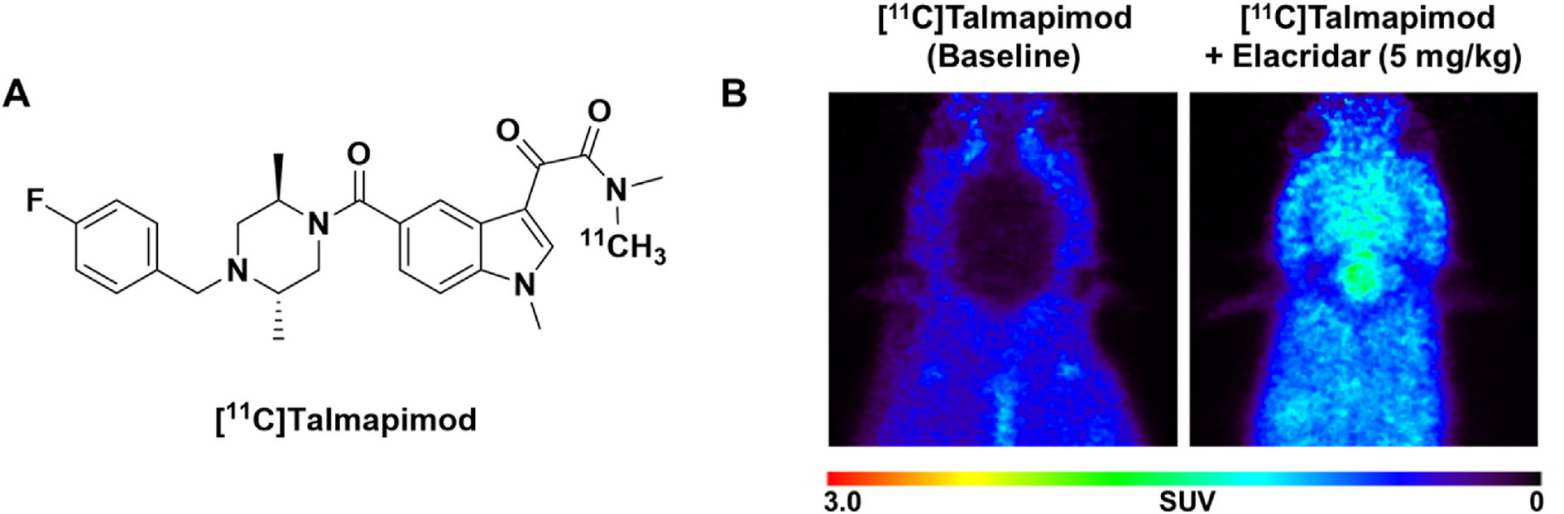
(A) Structure of [^11^C]talmapimod. (B) [^11^C]Talmapimod PET images (average SUV 0–90 ​min) in healthy rodent at baseline and after P-glycoprotein drug efflux transporter inhibition with 5 ​mg/kg elacridar administered 30 ​min prior to radiotracer injection. Adapted with permission from Chassé M et al. In vitro and in vivo evaluation of [^11^C]M4K2127 for PET imaging activin receptor-like kinase 2. Nucl Med Biol 2023; 126: 108677.

To assess the brain uptake of intravenously administered talmapimod, the structure was recently radiolabeled in our labs with carbon-11 (Fig. 7A) and evaluated preclinically in rodents [70]. PET imaging with [^11^C]talmapimod revealed low baseline brain uptake (0.2 SUV); however, disrupting P-glycoprotein (P-gp) drug efflux transporter activity through pretreatment with elacridar enabled [^11^C]talmapimod to pass the blood–brain barrier (>1.0 SUV) [71] (Fig. 7B). A subsequent MDCK-MDR1 assay confirmed the drug efflux activity of this compound (efflux ratio of 10.2), suggesting that talmapimod is unlikely to exhibit large degrees of CNS exposure in humans. Even after surpassing the observed drug efflux activity, [^11^C]talmapimod exhibited poor specific binding in vivo and in vitro (<15 ​%), suggesting that the degree of drug-to-target interactions under neurophysiological conditions may not be ideal for therapeutic purposes. In this example, PET was a useful investigational tool to determine the potential of repurposing a known compound for CNS conditions. Through preclinical PET imaging studies, talmapimod could reasonably be discounted as a potential neurotherapeutic because of discovered drug efflux activity and poor specific binding.

### Amyloid beta plaques

The neurological hallmarks of Alzheimer's disease (AD) are the presence of amyloid beta plaques, hyperphosphorylated tau aggregates, and neuronal loss – otherwise known as the ‘A/T/N’ criteria (amyloid/tau/neurodegeneration) – although the addition of other relevant biomarkers is an active area of research [[72], [73], [74], [75]]. While multi-tracer approaches are needed for PET imaging of AD and related dementias [[76], [77], [78], [79], [80]], here we will focus on how PET has been useful for neurotherapeutic development related to amyloid plaques. The amyloid hypothesis suggests that amyloid plaque related toxicity is the primary cause of neuronal loss which results in AD progression [81,82]. Over the past several decades, many failed attempts have been made to target amyloid plaque formation and/or clearance as a means of treating AD. The lack of success of these amyloid-targeting therapies are hypothesized to arise from inappropriate in vivo characteristics, including lack of brain exposure, poor target engagement, lack of specificity, and/or improper patient selection for clinical trials [82].

**Fig. 8:**
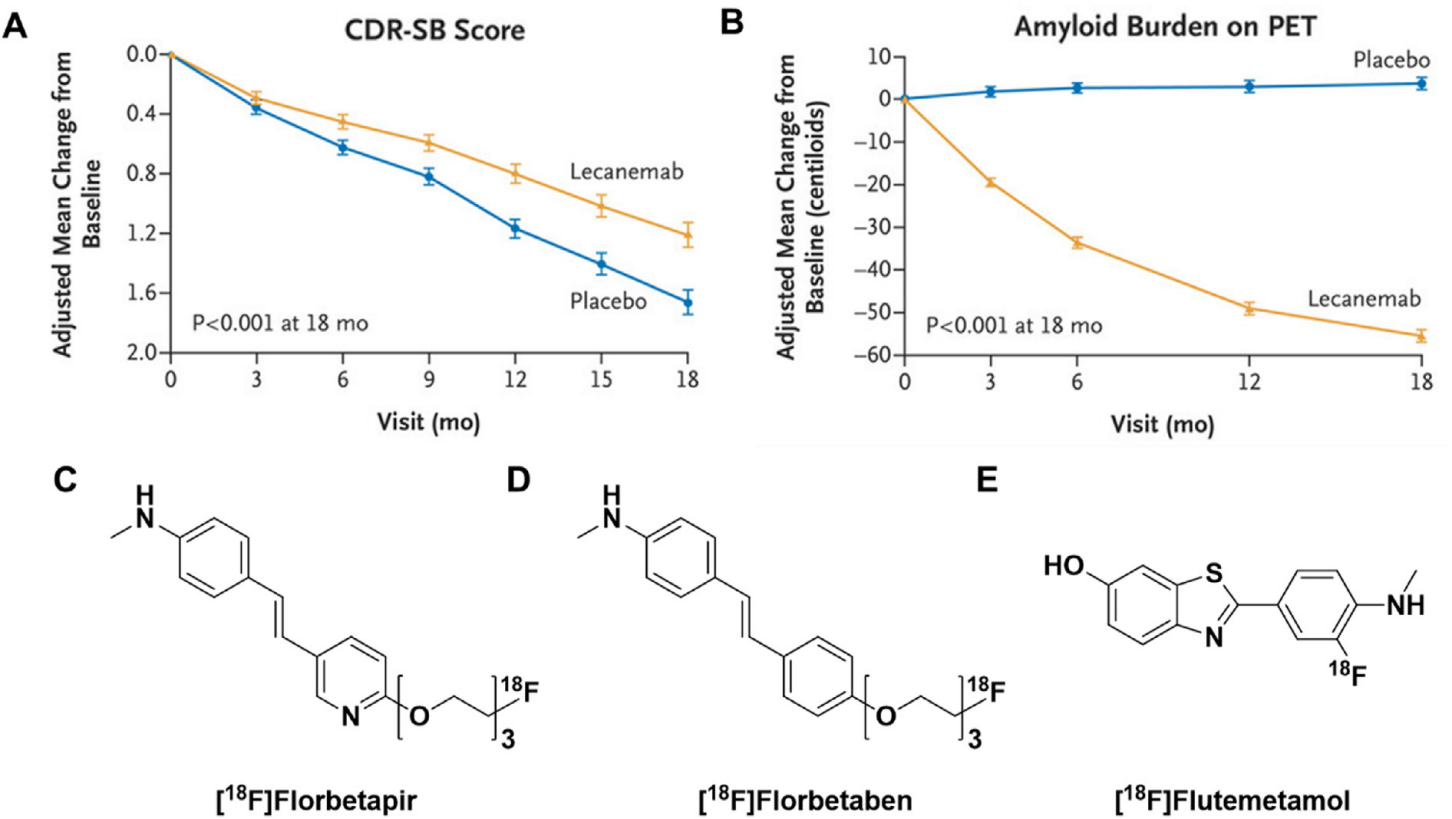
(A) Change in the score on the Clinical Dementia Rating (CDR)–Sum of Boxes (CDR-SB) from baseline in an 18-month, multicenter, double-blind, phase 3 trial of lecanemab in patients with early AD versus placebo group. (B) Adjusted mean change in amyloid burden in treatment versus placebo groups as measured by amyloid PET using (C) [^18^F]florbetapir (Amyvid®), (D) [^18^F]florbetaben (Neuraceq®), or (E) [^18^F]flutemetamol (Vizamyl®). Graphical data adapted with permission from van Dyck et al. Lecanemab in Early Alzheimer's Disease. N Engl J Med 2023; 388:9–21.

Recently two monoclonal anti-amyloid antibodies, lecanemab (Leqembi®) and aducanumab (Aduhelm®), were approved in the United States through the Food and Drug Administration (FDA) accelerated approval mechanism. Amyloid PET imaging has played an instrumental role in the research and approval of these disease modifying therapies and continues to play a pivotal role in ongoing research in this area [[83], [84], [85], [86], [87], [88]]. The radiotracers primarily used in this space are the three FDA-approved amyloid PET radiopharmaceuticals, Amyvid® (AV-45; [^18^F]fluorbetapir, 2012), Neuraceq® (AV-1; BAY-94-9172, [^18^F]florbetaben, 2014), and Vizamyl® (GE-067, [^18^F]flutemetamol, 2013) (Fig. 8).

In many trials, amyloid PET imaging was used as part of the inclusion criterion to ensure that patients enrolled in the clinical trial expressed the target in sufficient quantities to test the therapeutic potential of these antibodies. Moreover, PET imaging permits researchers to directly quantify biomarker expression in the brain instead of having to rely solely on peripheral measures, like protein levels in blood plasma or cerebral spinal fluid, to estimate brain amyloid plaque levels [89,90]. As a result, changes in brain amyloid can be used as a measure of treatment response, and further correlated with other measures to support claims of disease modification through slowing of clinical decline. It should be noted that, while the claims of efficacy, clinical utility of some anti-amyloid treatments, and dosing regimens remain the subjects of much debate [[91], [92], [93]], there is no question that these antibodies do lead to a clear reduction in brain amyloid plaque load (Fig. 8A,B). Ultimately, the accelerated approvals of both lecanemab and aducanumab relied heavily on the integral use of PET imaging in patient selection, data collection, and treatment response monitoring.

**Fig. 9:**
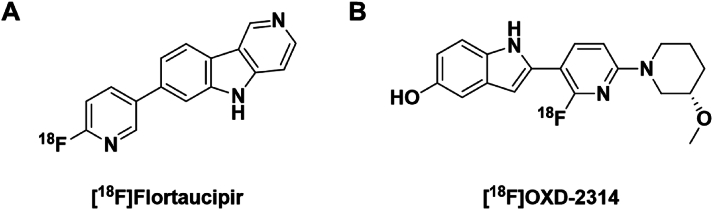
(A) Structure of [^18^F]Flortaucipir (Tauvid™, [^18^F]AV-1451, [^18^F]T807). (B) Structure of [^18^F]OXD-2314.

While amyloid has paved the way for clinical applications of PET imaging for neurotherapeutic development in AD, the radiotracer Tauvid™ (a.k.a. [^18^F]flortaucipir, [^18^F]AV-1451, [^18^F]T807) was approved by the FDA for clinical imaging of aggregated tau in 2020 (Fig. 9A). This approval has opened the door for clinical tau burden imaging, with tau PET radiopharmaceuticals are now actively being used to de-risk neurotherapeutic development in AD programs [94]. However, it is important to recognize that tau aggregates take different forms depending on the disease. Many of the well-established tau PET tracers were developed for AD and demonstrate limited utility for imaging non-AD tauopathies (e.g. progressive supranuclear palsy, corticobasal degeneration, chronic traumatic encephalopathy, Pick's disease, etc). Novel tau PET agents developed for non-AD tauopathies are actively being developed to satisfy these gaps, including [^18^F]OXD-2314, which we recently translated for first-in-human studies (Fig. 9B) [95].

PET imaging has been widely used as a tool for the development and evaluation of neurotherapeutics. In this article, we showcase a few recent examples of the ways that PET imaging can aid drug discovery and development programs. For instance, recent efforts in imaging psychiatric disease have demonstrated how radiotracers can be employed for studying biomarker function, performing target occupancy and engagement studies, and determining appropriate dosing regimens (e.g. [^11^C]Cimbi-36, [^11^C] AS2471907, [^18^F]AS2471907). Novel radiotracers developed for neuro-oncology and neurodegeneration emphasized the utility of PET for assessing drug pharmacokinetics and pharmacodynamics, including brain permeability, drug efflux effects, and metabolism (e.g. [^11^C]AZD4747, [^11^C]M4K2127, [^11^C]Talmapimod). In later stages of neurotherapeutic development, PET imaging can enable effective patient population selection, and direct quantification of biomarker changes in response to treatment, as was shown with FDA-approved radiopharmaceuticals for imaging Alzheimer's disease. From biomarker validation through to clinical studies, PET imaging is a versatile tool that can be used to support and inform CNS drug development programs.

## Author Contributions

M.C.: conceptualization, writing, figures, and editing. N.V.: conceptualization, supervision, and editing. Both authors reviewed the manuscript.

## Declaration of competing interest

The authors declare the following financial interests/personal relationships which may be considered as potential competing interests:Neil Vasdev reports financial support was provided by Azrieli Foundation and the Canada Research Chairs Program. Melissa Chasse reports financial support was provided by Canadian Institutes of Health Research for a Canada Graduate Scholarships. If there are other authors, they declare that they have no known competing financial interests or personal relationships that could have appeared to influence the work reported in this paper.

## References

[1] Steinmetz J.D., Seeher K.M., Schiess N., Nichols E., Cao B., Servili C. (2024). Global, regional, and national burden of disorders affecting the nervous system, 1990–2021: a systematic analysis for the Global Burden of Disease Study 2021. Lancet Neurol.

[2] Ferrari A.J., Santomauro D.F., Mantilla Herrera A.M., Shadid J., Ashbaugh C., Erskine H.E. (2022). Global, regional, and national burden of 12 mental disorders in 204 countries and territories, 1990–2019: a systematic analysis for the Global Burden of Disease Study 2019. Lancet Psychiatr.

[3] Kesselheim A., Hwang T., Franklin J. (2015). Two decades of new drug development for central nervous system disorders. Nat Rev Drug Discov.

[4] Wegener G., Rujescu D. (2013). The current development of CNS drug research. Int J Neuropsychopharmacol.

[5] Lindsley C.W. (2013). Statistics for global prescription medications: CNS therapeutics maintain a leading position among small molecule therapeutics. ACS Chem Neurosci.

[6] Hughes J.P., Rees S., Kalindjian S.B., Philpott K.L. (2010). Principles of early drug discovery. Br J Pharmacol.

[7] Austin D., Hayford T., Kile J., Nelson L., Topoleski J., Adams C. (2021).

[8] Danon J.J., Reekie T.A., Kassiou M. (2019). Challenges and opportunities in central nervous system drug discovery. Trends Chem.

[9] Nance E., Pun S.H., Saigal R., Sellers D.L. (2021). Drug delivery to the central nervous system. Nat Rev Mater.

[10] Lindberg A., Chassé M., Varlow C., Pees A., Vasdev N. (2023). Strategies for designing novel positron emission tomography (PET) radiotracers to cross the blood–brain barrier. J Label Compd Radipharm.

[11] Pangalos M.N., Schechter L.E., Hurko O. (2007). Drug development for CNS disorders: strategies for balancing risk and reducing attrition. Nat Rev Drug Discov.

[12] Hay M., Thomas D.W., Craighead J.L., Economides C., Rosenthal J. (2014). Clinical development success rates for investigational drugs. Nat Biotechnol.

[13] Wong C.H., Siah K.W., Lo A.W. (2019). Estimation of clinical trial success rates and related parameters. Biostatistics.

[14] Harrison R.K. (2016). Phase II and phase III failures: 2013–2015. Nat Rev Drug Discov.

[15] Schlander M., Hernandez-Villafuerte K., Cheng C.Y., Mestre-Ferrandiz J., Baumann M. (2021). How much does it cost to research and develop a new drug? A systematic review and assessment. Pharmacoeconomics.

[16] Gunn R.N., Summerfield S.G., Salinas C.A., Read K.D., Guo Q., Searle G.E. (2012). Combining PET biodistribution and equilibrium dialysis assays to assess the free brain concentration and BBB transport of CNS drugs. J Cerebr Blood Flow Metabol.

[17] Etkin A., Powell J., Savitz A.J. (2024). Opportunities for use of neuroimaging in de-risking drug development and improving clinical outcomes in psychiatry: an industry perspective. Neuropsychopharmacol.

[18] Nutt D.J., Erritzoe D., Carhart-Harris R. (2020). Psychedelic psychiatry's brave new world. Cell.

[19] Yao Y., Guo D., Lu T.S., Liu F.L., Huang S.H., Diao M.Q. (2024). Efficacy and safety of psychedelics for the treatment of mental disorders: a systematic review and meta-analysis. Psychiatr Res.

[20] Andersen K.A.A., Carhart-Harris R., Nutt D.J., Erritzoe D. (2020). Therapeutic effects of classic serotonergic psychedelics: a systematic review of modern-era clinical studies. Acta Psychiatr Scand.

[21] Wallach J., Cao A.B., Calkins M.M., Heim A.J., Lanham J.K., Bonniwell E.M. (2023). Identification of 5-HT2A receptor signaling pathways associated with psychedelic potential. Nat Commun.

[22] Wall M.B., Harding R., Zafar R., Rabiner E.A., Nutt D.J., Erritzoe D. (2023). Neuroimaging in psychedelic drug development: past, present, and future. Mol Psychiatr.

[23] Cumming P., Scheidegger M., Dornbierer D., Palner M., Quednow B.B., Martin-Soelch C. (2021). Molecular and functional imaging studies of psychedelic drug action in animals and humans. Molecules.

[24] Ettrup A., Hansen M., Santini M.A., Paine J., Gillings N., Palner M. (2010). Radiosynthesis and in vivo evaluation of a series of substituted 11C-phenethylamines as 5-HT2A agonist PET tracers. Eur J Nucl Med Mol Imag.

[25] Finnema S.J., Stepanov V., Ettrup A., Nakao R., Amini N., Svedberg M. (2014). Characterization of [^11^C]Cimbi-36 as an agonist PET radioligand for the 5-HT2A and 5-HT2C receptors in the nonhuman primate brain. Neuroimage.

[26] Ettrup A., Svarer C., McMahon B., da Cunha-Bang S., Lehel S., Møller K. (2016). Serotonin 2A receptor agonist binding in the human brain with [^11^C]Cimbi-36: test-retest reproducibility and head-to-head comparison with the antagonist [^18^F]altanserin. Neuroimage.

[27] Madsen M.K., Fisher P.M., Burmester D., Dyssegaard A., Stenbæk D.S., Kristiansen S. (2019). Psychedelic effects of psilocybin correlate with serotonin 2A receptor occupancy and plasma psilocin levels. Neuropsychopharmacol.

[28] Becker A.M., Klaiber A., Holze F., Istampoulouoglou I., Dthaler U., Varghese N. (2023). Ketanserin reverses the acute response to LSD in a randomized, double-blind, placebo-controlled, crossover study in healthy participants. Int J Neuropsychopharmacol.

[29] Preller K.H., Herdener M., Pokorny T., Liechti M.E., Seifritz E., Vollenweider F.X. (2017). The fabric of meaning and subjective effects in LSD-induced States depend on serotonin 2A receptor activation. Curr Biol.

[30] Holze F., Vizeli P., Ley L., Muller F., Dolder P., Stocker M. (2021). Acute dose-dependent effects of lysergic acid diethylamide in a double-blind placebo-controlled study in healthy subjects. Neuropsychopharmacol.

[31] Vollenweider F.X., Vollenweider-Scherpenhuyzen M.F., Babler A., Vogel H., Hell D. (1998). Psilocybin induces schizophrenia-like psychosis in humans via a serotonin-2 agonist action. Neuroreport.

[32] Holze F., Madsen M.K., Svarer C., Gillings N., Stenbaek D.S., Rudin D. (2024). Ketanserin exhibits dose- and concentration-proportional serotonin 2A receptor occupancy in healthy individuals: relevance for psychedelic research. Eur Neuropsychopharmacol.

[33] Chapman K., Holmes M., Secki J. (2013). 11β-Hydroxysteroid dehydrogenases: intracellular gate-keepers of tissue glucocorticoid action. Physiol Rev.

[34] Wyrwoll C.S., Holmes M.C., Secki J.R. (2011). 11β-Hydroxysteroid dehydrogenases and the brain: from zero to hero, a decade of progress. Front Neuroendocrinol.

[35] Bhatt S., Hillmer A.T., Rusowicz A., Nabulsi N., Matuskey D., Angarita G.A. (2021). Imaging brain cortisol regulation in PTSD with a target for 11β-hydroxysteroid dehydrogenase type 1. J Clin Invest.

[36] Verplaetse T.L., Hillmer A.T., Bhatt S., Rusowicz A., Li S., Nabulsi N. (2024). Imaging a putative marker of brain cortisol regulation in alcohol use disorder. Neurobiol Stress.

[37] Yehuda R., Teicher M.H., Trestman R.L., Levengood R.A., Siever L.J. (1996). Cortisol regulation in posttraumatic stress disorder and major depression: a chronobiological analysis. Biol Psychiatr.

[38] Bini J., Bhatt S., Hillmer A.T., Gallezot J.D., Nabulsi N., Pracitto R. (2020). Body mass index and age effects on brain 11β-hydroxysteroid dehydrogenase type 1: a positron emission tomography study. Mol Imag Biol.

[39] Bellaire S., Walzer M., Wang T., Krauwinkel W., Yuan N., Marek G.J. (2019). Safety, pharmacokinetics, and pharmacodynamics of ASP3662, a novel 11β-hydroxysteroid dehydrogenase type 1 inhibitor, in healthy young and elderly subjects. Clin Transl Sci.

[40] Wenping L., Joshi A., Zhizhen Z., O'Malley S.S., Miller P.J., Riffel K. (2011). Radiosynthesis and evaluation of an 11-β hydroxy- steroid dehydrogenase-1 (11β-HSD1) PET ligand in rhesus monkey. J Label Compd Radiopharm.

[41] Iwashita A., Fushiki H., Fujita Y., Murakami Y., Noda A. (2016). Discovery of a novel radioligand [^11^C]AS2471907 for PET imaging of the brain 11β-HSD1. J Nucl Med.

[42] Baum E., Zhang W., Li S., Cai Z., Holden D., Huang Y. (2019). A novel ^18^F-labeled radioligand for positron emission tomography imaging of 11β-hydroxysteroid dehydrogenase (11β-HSD1): synthesis and preliminary evaluation in nonhuman primates. ACS Chem Neurosci.

[43] Bhatt S., Nabulsi N., Li S., Cai Z., Matuskey D., Bini J. (2020). First in-human PET study and kinetic evaluation of [^18^F]AS2471907 for imaging 11β-hydroxysteroid dehydrogenase type 1. J Cerebr Blood Flow Metabol.

[44] Siegel R.L., Miller K.D., Wagle N.S., Jemal A. (2023). Cancer statistics, 2023. CA Cancer J Clin.

[45] Liu Y.P., Zheng C.C., Huang Y.N., He M.L., Xu W.W., Li B. (2021). Molecular mechanisms of chemo- and radiotherapy resistance and the potential implications for cancer treatment. Media Commun.

[46] Huang L., Guo Z., Wang F., Fu L. (2021). KRAS mutation: from undruggable to druggable in cancer. Signal Transduct Targeted Ther.

[47] Kettle J.G., Bagal S.K., Barratt D., Bodnarchuk M.S., Boyd S., Braybrooke E. (2023). Discovery of AZD4747, a potent and selective inhibitor of mutant GTPase KRASG12C with demonstrable CNS penetration. J Med Chem.

[48] Pike K.G., Hunt T.A., Barlaam B., Benstead D., Cadogan E., Chen K. (2024). Identification of novel, selective ataxia-telangiectasia mutated kinase inhibitors with the ability to penetrate the blood–brain barrier: the discovery of AZD1390. J Med Chem.

[49] Du S., Liang Q., Shi J. (2024). Progress of ATM inhibitors: opportunities and challenges. Eur J Med Chem.

[50] Jucaite A., Stenkrona P., Cselenyi Z., De Vita A., Buil-Bruna N., Varnas K. (2021). Brain exposure of the ATM inhibitor AZD1390 in humans—a positron emission tomography study. Neuro Oncol.

[51] Sanai N., Desai S., Margaryan T., Lo Cascio C., Elliot M., Molloy J. (2023). 505MO A phase 0/Ib study of AZD1390 plus radiotherapy in recurrent glioblastoma patients. Ann Oncol.

[52] Loomans H.A., Andl C.D. (2016). Activin receptor-like kinases: a diverse family playing an important role in cancer. Am J Cancer Res.

[53] Valer J.A., Sánchez-de-Diego C., Pimenta-Lopes C., Rosa J.L., Ventura F. (2019). ACVR1 function in health and disease. Cells.

[54] Villanueva M. (2014). *ACVR1* mutations—a key piece in paediatric diffuse glioma. Nat Rev Clin Oncol.

[55] Carvalho D., Taylor K.R., Olaciregui N.G., Molinari V., Clarke M., Mackay A. (2019). ALK2 inhibitors display beneficial effects in preclinical models of ACVR1 mutant diffuse intrinsic pontine glioma. Commun Biol.

[56] Rooney L., Jones C. (2021). Recent advances in ALK2 inhibitors. ACS Omega.

[57] Ensan D., Smil D., Zepeda-Velázquez C.A., Panagopoulos D., Wong J.F., Williams E.P. (2020). Targeting ALK2: an open science approach to developing therapeutics for the treatment of diffuse intrinsic pontine glioma. J Med Chem.

[58] Smil D., Wong J.F., Williams E.P., Adamson R.J., Howarth A., McLeod D.A. (2020). Leveraging an open science drug discovery model to develop CNS-penetrant ALK2 inhibitors for the treatment of diffuse intrinsic pontine glioma. J Med Chem.

[59] González-Álvarez H., Ensan D., Xin T., Wong J.F., Zepeda-Velázquez C.A., Cros J. (2024). Discovery of conformationally constrained ALK2 inhibitors. J Med Chem.

[60] Davis A.J., Brooijmans N., Brubaker J.D., Stevison F., LaBranche T.P., Albayya F. (2024). An ALK2 inhibitor, BLU-782, prevents heterotopic ossification in a mouse model of fibrodysplasia ossificans progressiva. Sci Transl Med.

[61] Němec V., Remeš M., Beňovský P., Böck M.C., Šranková E., Wong J.F. (2024). Discovery of two highly selective structurally orthogonal chemical probes for activin receptor-like kinases 1 and 2. J Med Chem.

[62] Murrell E., Tong J., Smil D., Kiyota T., Aman A.M., Isaac M.B. (2021). Leveraging open science drug development for PET: preliminary neuroimaging of ^11^C-labeled ALK2 inhibitors. ACS Med Chem Lett.

[63] Chassé M., Murrell E., Tong J., Vasdev N. (2023). In vitro and in vivo evaluation of [^11^C]M4K2127 for PET imaging activin receptor-like kinase 2. Nucl Med Biol.

[64] Pike V.W. (2009). PET radiotracers: crossing the blood-brain barrier and surviving metabolism. Trends Pharmacol Sci.

[65] Canovas B., Nebreda A.R. (2021). Diversity and versatility of p38 kinase signalling in health and disease. Nat Rev Mol Cell Biol.

[66] Bachstetter A.D., Van Eldik L.J. (2010). The p38 MAP kinase family as regulators of proinflammatory cytokine production in degenerative diseases of the CNS. Aging Dis.

[67] Valipour M., Mohammadi M., Valipour H. (2024). CNS-active p38α MAPK inhibitors for the management of neuroinflammatory diseases: medicinal chemical properties and therapeutic capabilities. Mol Neurobiol.

[68] Siegel D.S., Krishnan A., Lonial S., Chatta G., Alsina M., Jagannath S. (2006). Phase II trial of SCIO-469 as monotherapy (M) or in combination with bortezomib (MB) in relapsed refractory multiple myeloma (MM). Blood.

[69] Genovese M.C., Cohen S.B., Wofsy D., Weinblatt M.E., Firestein G.S., Brahn E. (2011). A 24-week, randomized, double-blind, placebo-controlled, parallel group study of the efficacy of oral SCIO-469, a p38 mitogen-activated protein kinase inhibitor, in patients with active rheumatoid arthritis. J Rheumatol.

[70] Chassé M., Vasdev N. (2023). Synthesis and preclinical positron emission tomography imaging of the p38 MAPK inhibitor [^11^C]talmapimod: effects of drug efflux and sex differences. ACS Chem Neurosci.

[71] Hyafil F., Vergely C., Du Vignaud P., Grand-Perret T. (1993). In vitro and in vivo reversal of multidrug resistance by GF120918, an acridonecarboxamide derivative. Cancer Res.

[72] Jack C.R., Bennett D.A., Blennow K., Carrillo M.C., Feldman H.H., Frisoni G.B. (2016). A/T/N: an unbiased descriptive classification scheme for Alzheimer disease biomarkers. Neurology.

[73] Jack C.R., Bennett D.A., Blennow K., Carrillo M.C., Dunn B., Haeberlein S.B. (2018). NIA-AA Research Framework: toward a biological definition of Alzheimer's disease. Alzheimers Dement.

[74] Hampel H., Cummings J., Blennow K., Gao P., Jack C.R., Vergallo A. (2021). Developing the ATX(N) classification for use across the Alzheimer disease continuum. Nat Rev Neurol.

[75] Imbimbo B.P., Watling M., Imbibo C., Nistico R. (2023). Plasma ATN(I) classification and precision pharmacology in Alzheimer's disease. Alzheimers Dement.

[76] Kallinen A., Kassiou M. (2022). Tracer development for PET imaging of proteinopathies. Nucl Med Biol.

[77] Perani D., Iaccarino L., Lammertsma A., Windhorst A.D., Edison P., Boellaard R. (2019). A new perspective for advanced positron emission tomography–based molecular imaging in neurodegenerative proteinopathies. Alzheimer's Dementia.

[78] Villemagne V.L., Doré V., Burnham S.C., Masters C.L., Rowe C.C. (2018). Imaging tau and amyloid-β proteinopathies in Alzheimer disease and other conditions. Nat Rev Neurol.

[79] Chassé M., Vasdev N. (2024). Emerging targets for positron emission tomography imaging in proteinopathies. Npj Imaging.

[80] d'Orchymont F., Narvaez A., Raymond R., Sachdev P., Charil A., Krause S. (2024). In vitro evaluation of PET radiotracers for imaging synaptic density, the acetylcholine transporter, AMPA-tarp-γ8 and muscarinic M4 receptors in Alzheimer's disease. Am J Nucl Med Mol Imaging.

[81] Selkoe D.J., Hardy J. (2016). The amyloid hypothesis of Alzheimer's disease at 25 years. EMBO Mol Med.

[82] Karran E., De Strooper B. (2022). The amyloid hypothesis in Alzheimer disease: new insights from new therapeutics. Nat Rev Drug Discov.

[83] Cummings J. (2023). Anti-amyloid monoclonal antibodies are transformative treatments that redefine alzheimer's disease therapeutics. Drugs.

[84] Sevigny J., Chiao P., Bussière T., Weinreb P.H., Williams L., Maier M. (2016). The antibody aducanumab reduces Aβ plaques in Alzheimer's disease. Nature.

[85] Mintun M.A., Lo A.C., Evans C.D., Wessels A.M., Ardayfio P.A., Andersen S.W. (2021). Donanemab in early Alzheimer's disease. N Engl J Med.

[86] Swanson C.J., Zhang Y., Dhadda S., Wang J., Kaplow J., Lai R.Y.K. (2021). A randomized, double-blind, phase 2b proof-of-concept clinical trial in early Alzheimer's disease with lecanemab, an anti-Aβ protofibril antibody. Alz Res Therapy.

[87] Rinne J.O., Brooks D.J., Rossor M.N., Fox N.C., Bullock R., Klunk W.E. (2010). 11C-PiB PET assessment of change in fibrillar amyloid-β load in patients with Alzheimer's disease treated with bapineuzumab: a phase 2, double-blind, placebo-controlled, ascending-dose study. Lancet Neurol.

[88] van Dyck C.H., Swanson C.J., Aisen P., Bateman R.J., Chen C., Gee M. (2023). Lecanemab in early Alzheimer's disease. N Engl J Med.

[89] Apostolova L.G., Hwang K.S., Avila D., Elashoff D., Kohannim O., Teng E. (2015). Brain amyloidosis ascertainment from cognitive, imaging, and peripheral blood protein measures. Neurology.

[90] Cohen A.D., Landau S.M., Snitz B.E., Klunk W.E., Blennow K., Zetterberg H. (2019). Fluid and PET biomarkers for amyloid pathology in Alzheimer's disease. Mol Cell Neurosci.

[91] Knopman D.S., Jones D.T., Greicius M.D. (2020). Failure to demonstrate efficacy of aducanumab: an analysis of the EMERGE and ENGAGE trials as reported by Biogen, December 2019. Alzheimer's Dementia.

[92] Mullard A. (2021). FDA approval for Biogen's aducanumab sparks Alzheimer disease firestorm. Nat Rev Drug Discov.

[93] Alexander G.C., Knopman D.S., Emerson S.S., Ovbiagele B., Kryscio R.J., Perlmutter J.S. (2021). Revisiting FDA approval of aducanumab. N Engl J Med.

[94] Therriault J., Schindler S.E., Salvadó G., Pascoal T.A., Benedet A.L., Ashton N.J. (2024). Biomarker-based staging of Alzheimer disease: rationale and clinical applications. Nat Rev Neurol.

[95] Lindberg A., Murrell E., Tong J., Mason N.S., Sohn D., Sandell J. (2024). Ligand-based design of [^18^F]OXD-2314 for PET imaging in non-Alzheimer’s disease tauopathies. Nat Commun.

